# Continuous cropping system altered soil microbial communities and nutrient cycles

**DOI:** 10.3389/fmicb.2024.1374550

**Published:** 2024-04-12

**Authors:** Mengjiao Ding, Huaxin Dai, Yi He, Taibo Liang, Zhen Zhai, Shixiang Zhang, Binbin Hu, Heqing Cai, Bin Dai, Yadong Xu, Yanling Zhang

**Affiliations:** ^1^Zhengzhou Tobacco Research Institute of CNTC, Zhengzhou, China; ^2^College of Tobacco Science of Guizhou University, Guiyang, China; ^3^Guizhou Provincial Key Laboratory for Tobacco Quality, College of Tobacco Science, Guizhou University, Guiyang, China; ^4^School of Agricultural Sciences, Zhengzhou University, Zhengzhou, China; ^5^Guizhou Tobacco Company Bijie Region Tobacco Company, Bijie, China; ^6^Yunnan Academy of Tobacco Agricultural Sciences, Kunming, China

**Keywords:** continuous-cropping system, environmental factors, microbial community, nutrient cycling, rhizospheric soil

## Abstract

Understanding the response of microbial communities and their potential functions is essential for sustainability of agroecosystems under long-term continuous cropping. However, limited research has focused on investigating the interaction between soil physicochemical factors and microbial community dynamics in agroecosystems under long-term continuous cropping. This study probed into the physicochemical properties, metabolites, and microbial diversity of tobacco rhizosphere soils cropped continuously for 0, 5, and 20 years. The relative abundance of bacterial genera associated with nutrient cycling (e.g., *Sphingomonas*) increased while potential plant pathogenic fungi and beneficial microorganisms showed synergistic increases with the duration of continuous cropping. Variations in soil pH, alkeline nitrogen (AN) content, and soil organic carbon (SOC) content drove the shifts in soil microbial composition. Metabolites such as palmitic acid, 3-hydroxypropionic acid, stearic acid, and hippuric acid may play a key role in soil acidification. Those results enhance our ability to predict shifts in soil microbial community structure associated with anthropogenic continuous cropping, which can have long-term implications for crop production.

## Highlights

– Soil microbiome structure had different changes among different periods of continuous cropping.
– Soil pH, AN, and SOC were the key environmental factors contributing to soil microbial community structure changes.
– Pathogenic fungi disrupt the nutrient cycling in agroecosystems under continuous cropping practices.

## Introduction

Continuous cropping is a common farming practice in China (Zhang Y. Z. et al., 2022), involves growing the same crop on the same plot of land for extended periods with the aim of achieving high yields (Zhang S. et al., 2022). However, continuous cropping tobacco rather than reasonable crop rotation (tobacco-soybean-corn), often leads to severe continuous cropping obstacles, usually manifested as more frequent plant diseases epidemics and soil microbial community changes (Ma et al., 2022). Generally, continuous cropping accelerates soil acidification, thus promoting the outbreak of crop diseases (Li et al., 2017). The frequent occurrence of crop diseases reduces the quality of crops and even leads to crop failure in karst agroecosystems (Gao et al., 2019).

Tobacco has been extensively utilized as a model organism in numerous studies pertaining to plant biotechnology (Sierro et al., 2014). It is also an important crop widely planted in impoverished areas (Chen F. et al., 2018). Due to the limited arable land resources, tobacco is continuously grown on the same plots for over 20 years, leading to persistent challenges associated with continuous cropping and subsequent yield reductions (Matheri et al., 2023). Several studies have demonstrated that after 7 years of continuous cropping, tobacco plants could sustain wilt disease caused by soil-borne fungi (Niu et al., 2017). In addition, the modification of resident soil microbial flora is related to changes in soil physicochemical properties and soil enzymatic activities (Chen S. et al., 2018). Soil microorganisms are effective biological indicators of soil fertility as they play a significant role in agricultural production (Avidano et al., 2005; Chen W. et al., 2018; Yan et al., 2023). The diversity index of soil microbial community composition tends to decrease after long-term continuous cropping, reduce the number of beneficial microorganisms, and increase the abundance of pathogenic bacteria, thus reducing crop yields (Xiong et al., 2015; Gao et al., 2019). The previous studies have indicated a significant decline in the abundance of beneficial microorganisms with prolonged tobacco cropping (Maguire et al., 2020). Cultivation has been found to alter microbial communities, leading to a notable increase in *Fusarium* abundance. Consequently, urgent measures are required to effectively manage this pathogen in arable farmlands. However, limited research has focused on assessing soil health under long-term continuous tobacco cropping (Xiong et al., 2015). Therefore, safeguarding the soil agricultural micro-ecosystem is imperative for promoting optimal crop growth.

Crops secrete allelopathic substances such as secondary metabolites, which affect the soil environment and cause continuous cropping obstacles (Gerhards and Schappert, 2020). Soil microorganisms play a pivotal role in modulating the effects of these secondary metabolites, exerting both positive and negative influences on crop growth. Firstly, soil microorganisms decompose toxic compounds to reduce allelopathy (Jilani et al., 2008). Secondly, the breakdown of allelopathic residues by soil microorganisms can lead to the release of additional phytotoxins (Cipollini et al., 2012). Therefore, it is imperative to investigate the mechanisms underlying soil-borne diseases associated with continuous tobacco cropping.

By the time of writing, limited reports have elucidated the response of microbial communities to long-term continuous tobacco cropping. In this study, we employed Illumina sequencing and metabolomics to investigate the changes in soil microbial communities and the types of allelopathic substances under continuous cropping. The objectives encompassed (i) exploring the diversity and composition responses of microorganisms to varying durations of continuous cropping; (ii) evaluating the specific microbial taxa associated with the development of obstacles posed by continuous cropping; and (iii) predicting the variation tendency of karst agroecosystems and their responses to prolonged periods of continuous cropping.

## Materials and methods

### Soil collection

The experimental site was located in Weining experimental station, at Bijie City, Guizhou province, China (27°05′N, 103°51′E). The region has a typical karst plateau landform and a humid subtropical monsoon climate with a mean annual temperature of 18°C, an average sea level of 2,092 m, and annual precipitation of 926 mm. The main soil type was calcareous soil developed from limestone. Farmlands under continuous tobacco cropping for 5 and 20 years were selected for sampling as Y5 and Y20. Collected soil samples from cornfield adjacent to the sampling site Y5, where tobacco had never been planted, were used as the control (CK). Field management measures in CK were consistent with treatment groups to minimize any confusion caused by the effects of agricultural management practices on soil metabolites or microorganisms. The amount of fertilizer applied was 600 kg/ha of NPK compound fertilizer (N 15%, P_2_O_5_ 15%, and K_2_O 20%). All fertilizers were used as base fertilizer and applied to the soil at one time. Topdressing was performed every 30 days for two times with N: 20 kg·ha, P_2_O_5_: 20 kg·ha, and K_2_O: 50 kg·ha.

Three types of soil samples were collected from the plow layers (5–20 cm deep) in April 2021, labeled, respectively. In order to ensure the consistency of sampling time, samples were collected from three fields at the same day. Soil samples were collected from the surface of tobacco roots in plow layers (5–20 cm). Soil closely attached to the roots was washed using sterile water and pooled in a 50-mL Falcon tube as rhizosphere soil (Kui et al., 2021). In each field, samples were collected via a five-point sampling method to obtain representative samples. For each group, five soil samples were collected and combined to form a single sample, which was repeated three times. The above samples were divided into two parts. One was air-dried for physicochemical property analysis, and the other was stored at −80°C for DNA extraction.

### Analysis of soil basic properties

Soil pH levels were determined in suspensions with a soil-water (w/w) ratio of 1:2.5 (Ali et al., 2021). Soil organic carbon (SOC) contents were measured using the colorimetric method (Looft et al., 2012). Available phosphorus (AP) was extracted with sodium bicarbonate and analyzed using the molybdenum blue method (Pu et al., 2014). Available potassium (AK) contents were measured with ammonium acetate and assessed using flame photometry (Watanabe and Olsen, 1965). Available nitrogen (AN) was extracted using the alkaline hydrolysis diffusion method (Li et al., 2019).

### Analysis of soil microbial communities

Total DNA was extracted from soil samples (0.5 g each) using the TGuide Soil DNA Kit (Tiangen DP812, Beijing, China). The V3-V4 regions of the16S rRNA genes were amplified by the bacterial primers 338F (5′-ACTCCTACGGGAGGCAGCAG-3′) and 806R (5′-GGACTACHVGGGTWTCTAAT-3′). Universal primers ITS1F (5′-CTTGGTCATTTAGAGGAAGTAA-3′) and ITS2R (5′-GCTG CGTTCTTCATCGATGC-3′) were used to amplify fungal ITS1 region (Ward et al., 2017; Gu et al., 2022). We purified the PCR products using the Monarch DNA Gel Extraction Kit (T1020L, MA, United States). Then, the purified amplicons were employed for library construction using the Illumina Novaseq 6000 platform (Illumina, CA, United States) at Biomarker Technology Co., Ltd. (Beijing, China). The raw FASTQ files of the fungal and bacterial reads resulting from Illumina sequencing were filtered by Trimmomatic (version v0.33) and merged by Usearch (version v10) (Edgar, 2013; Bolger et al., 2014). These high-quality sequences were clustered into operational taxonomic units (OTUs) based on a 97% similarity threshold using UPARSE (Edgar et al., 2011). An averaged OTU table was generated by resampled OTU subsets under 90% of the minimum sequencing depth for further analysis to reduce the effects of different sequencing depths on the analyses (Liu S. et al., 2021). Bacterial and Fungal taxonomies were assessed against the 16S rRNA database (Silva v128) and the UNITE fungal ITS database (UNITE v8.0), respectively (Jiao et al., 2020; Chen et al., 2022). The alpha diversity metrics, beta diversity metrics, and other analyses were calculated using QIIME (Caporaso et al., 2011) and R packages (version 3.1.1) (R Core Team, 2019). All sequence data were deposited in NCBI Sequence Read Archive database under accession number PRJNA874867.

### Metabolite profiling

Each soil sample was frozen-crushed using a steel ball at 30 Hz for 1.5 min. Each sample (1,000 mg) was extracted using 1.0 mL of 70% aqueous methanol (AM) and 1 mL of ethyl acetate (EA) with 5 μL internal standard at 4°C. After centrifugation at 10,000 rpm at 4°C for 15 min, the supernatant was transferred into a 5-mL EP tube. After evaporation in a vacuum concentrator, 30 μL of ethoxyamination hydrochloride was added to the obtained product, and the mixture was then incubated at 80°C for 30 min and derivatized by 40 μL of BSTFA regent (1% TMCS, v/v) at 70°C for 1.5 h. All samples were adsorbed and filtrated before being subjected to analysis with a gas chromatograph (Agilent 7890, 30 m × 250 μm × 0.25 μm, J&W Scientific, Folsom, CA, United States) coupled with a time-of-flight mass spectrometer (GC-TOF-MS) (Kind et al., 2009).

### Statistical analysis

Based on the results of species annotations of all samples, the top 10 phyla and the top 35 genera were analyzed (relative abundance above 1%). Chao 1, Shannon, Simpson, and ACE indices were used to estimate the diversity of the microbial communities. Non-metric multidimensional scaling (NMDS) analysis was performed to investigate the structural variation of microbial communities across samples using Weighted uniFrac distance metrics (Looft et al., 2012). Principal component analysis (PCA) was also conducted based on OTU level compositional profiles (Ramette, 2007). The taxonomy compositions and abundances were visualized using MEGAN (Liu J. L. et al., 2021). Redundancy analyses (RDA) were used to investigate the relationships between samples and environmental variables (R: vegan package) (Oksanen et al., 2020). The heatmaps and Venn diagrams were generated using custom R scripts (Clauset et al., 2004). Bacterial functional potential was investigated with FAPROTAX database (Louca et al., 2016). Functional prediction analysis was carried out using FUNGuild database (Nilsson et al., 2019). All analyses were conducted on the BMK Cloud platform (Biomarker Technology Co., Ltd., Beijing, China; https://international.biocloud.net/zh/software/agriculture/) based on various R packages and workflow frameworks. A one-way ANOVA was performed to determine the significance of the differences of soil properties and microbial community structures between three treatments by SPSS. A *p* value <0.05 was considered to be statistically significant.

## Results

### Soil samples physicochemical properties

The physicochemical properties of soil samples after different continuous tobacco cropping durations are listed in Table 1. All samples were showed weak acidity. In addition, the soil pH level of Y20 decreased significantly. The SOC and AK contents in Y5 and Y20 were significantly lower than those of CK. However, the AP and AN content of Y20 were highest, reaching 33.75 and 162.32 mg kg^−1^, respectively.

**Table 1:** Soil properties with the duration of continuous cropping.

Samples	pH	SOC (g kg^−1^)	AP (mg kg^−1^)	AK (mg kg^−1^)	AN (mg kg^−1^)
CK	6.42 ± 0.07a	25.17 ± 1.16a	13.42 ± 5.15a	280.30 ± 21.42a	125.64 ± 21.99a
Y5	5.88 ± 0.02b	20.99 ± 1.84a	26.62 ± 9.99a	246.32 ± 15.61a	132.84 ± 11.62a
Y20	5.81 ± 0.03b	20.75 ± 0.37a	33.75 ± 7.10a	174.19 ± 2.69b	162.32 ± 7.15a

SOC, Soil organic carbon; AP, Available phosphorus; AK, Available potassium; AN, Alkaline hydrolysis nitrogen. CK: tobacco cropping for 0 years. Y5: continuous tobacco cropping for 5 years. Y20: continuous tobacco cropping for 20 years. Different letters in the same column indicate a significant difference among all treatments tested by one-way ANOVA (*p* < 0.05).

### Overall microbial community diversity

The 16S rRNA genes were sequenced to analyze the soil microbial community diversity under long-term continuous tobacco cropping. A total of 659,973 raw reads and 636,731 clean reads of bacteria were obtained, with an average length of 418 bp. As a result of chimeral filtration and QC, a total of 718,690 raw reads and 715,307 clean reads of fungi were obtained, with an average length of 237 bp. Through the analyses of alpha diversity, we found that the levels of bacterial richness tended to increase with the duration of continuous cropping. The richness of fungi reached the highest level after continuous cropping for 5 years, and then decreased gradually as the duration of continuous cropping increased further. Nevertheless, levels of soil fungal diversity tended to increased and then decreased, although no significant difference was found.

### Soil microbial community composition

**Figure 1:**
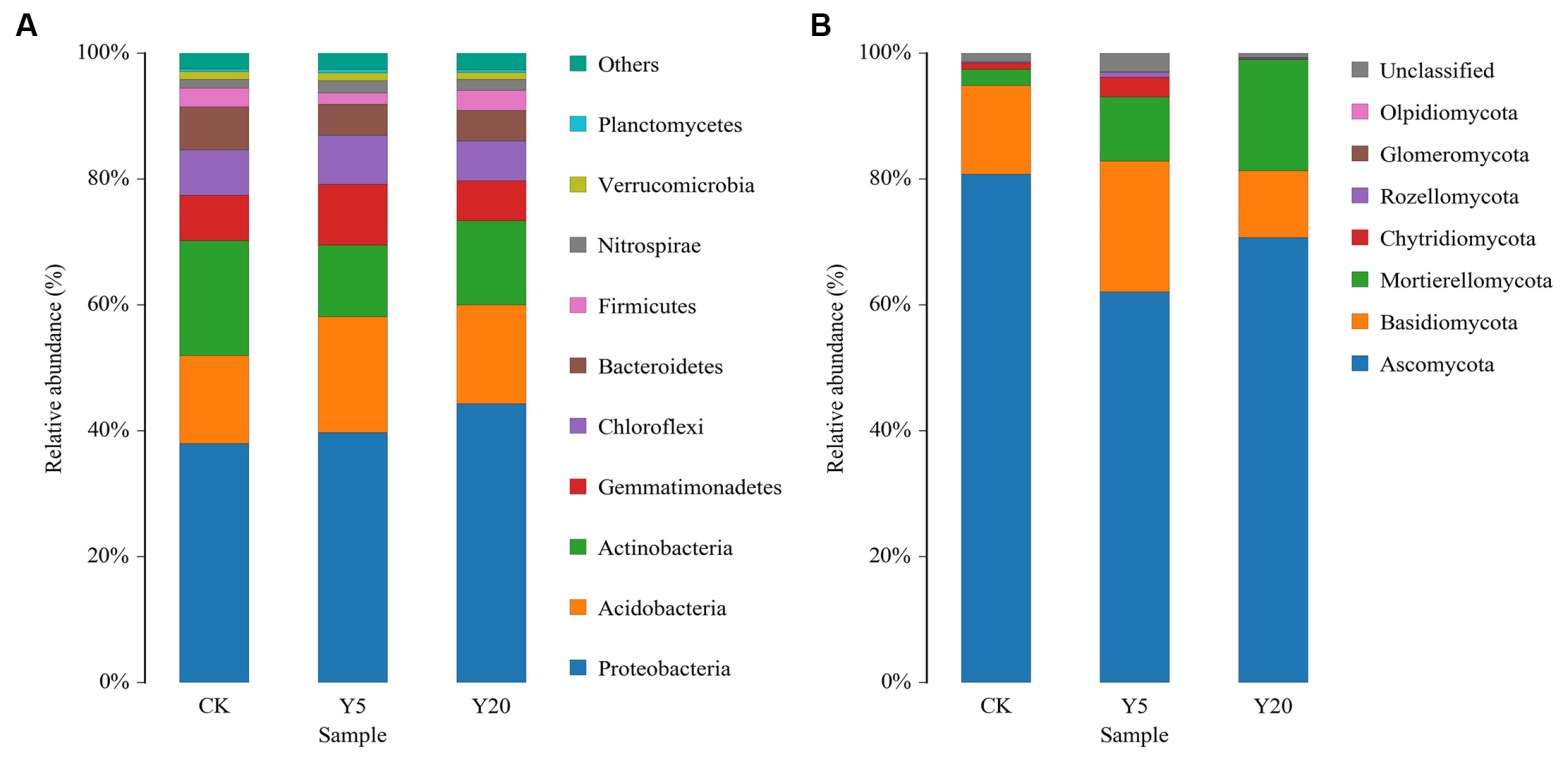
The phylum-level composition of **(A)** bacterial and **(B)** fungal communities. CK: tobacco cropping for 0 years. Y5: continuous tobacco cropping for 5 years. Y20: continuous tobacco cropping for 20 years.

The tobacco rhizosphere soils were home to 29 bacterial phyla, 81 classes, 183 orders, 303 families, and 565 genera. The dominant bacterial phyla in all samples included Proteobacteria, Acidobacteria, Actinobacteria, and Gemmatimonadetes, accounting for 77.44–9.74% of all sequences (Figure 1A). Proteobacteria dominated the rhizosphere soil, occupying 38.03–44.35% of all sequences, and their relative abundances in Y5 and Y20 were significantly higher than those in CK. The relative abundances of Acidobacteria, Gemmatimonadetes, Chloroflexi, Bacteroidetes, and Verrucomicrobia were higher in Y20 than in Y5. The relative abundances of dominant genera varied in the soil samples with different continuous cropping durations. The relative abundances of *Gemmatimonas*, *Gemmatimonadaceae*, and *Ramlibacter* in Y5 were significantly higher than that in CK and Y20. The relative abundances of *Sphingomonas*, *Massilia*, *Acidobacteriales*, *Burkholderiaceae*, *Xanthobacteraceae*, and *Gaiellales* in Y20 were significantly higher than in CK. Whereas the relative abundances of *Gemmatimonas*, *Micrococcaceae*, and *Micromonosporaceae* showed the opposite trend.

The tobacco rhizosphere soils were inhabited by seven fungal phyla, 22 classes, 57 orders, 118 families, and 202 genera. Ascomycota was the most abundant among with a relative abundance of 62.06–80.81%, followed by Basidiomycota (10.63 to 20.77%), Mortierellomycota (2.56 to 17.71%), and Chytridiomycota (0.74–9.56%) (Figure 1B). The relative abundance of Ascomycota in Y20 was significantly higher than that in Y5. However, the relative abundance of Mortierellomycota increased with the length of continuous cropping, with that in Y20 15.51% higher than that in CK. At the genus level, the relative abundances of *Mortierella*, *Byssochlamys*, and *Pseudopyrenochaeta* decreased and then increased with the duration of continuous cropping; in contrast, that of *Penicillium*, *Plectosphaerella*, *Aspergillus*, *Sebacina*, *Botryotinia*, and *Inocybe* increased first and then decreased. Among the top 15 genera, the relative abundances of *Cladosporium* (11.55%), *Epicoccum* (16.09%), *Vishniacozyma* (6.95%), and *Alternaria* (7.80%) in CK were higher than that in Y5 and Y20. Additionally, the relative abundances of *Mortierella* (17.70%), *Fusarium* (10.30%), *Chaetomium* (8.69%), and *Byssochlamys* (10.23%) in Y20 were higher than that in other samples.

### Differences in microbial communities

The principal component analysis (PCA) was used to analyze the community structure differences between groups (Supplementary Figure S1A). For bacteria, the first principle component (PC1) and the second principle component (PC2) contributed 37.63 and 23.04%, respectively. The Y20 samples were distinctive from the CK and Y5 samples. Y20 had the highest PC1 value, while Y5 had the highest PC2 value. CK had the lowest PC1 and PC2 values. For fungi, PC1 contributed 32.58%, and PC2 contributed 21.59% (Supplementary Figure S1B). Y20 had the highest PC1 value, and CK had the highest PC2 value. The Y5 and Y20 samples were highly similar and distinctive from the CK samples.

**Figure 2:**
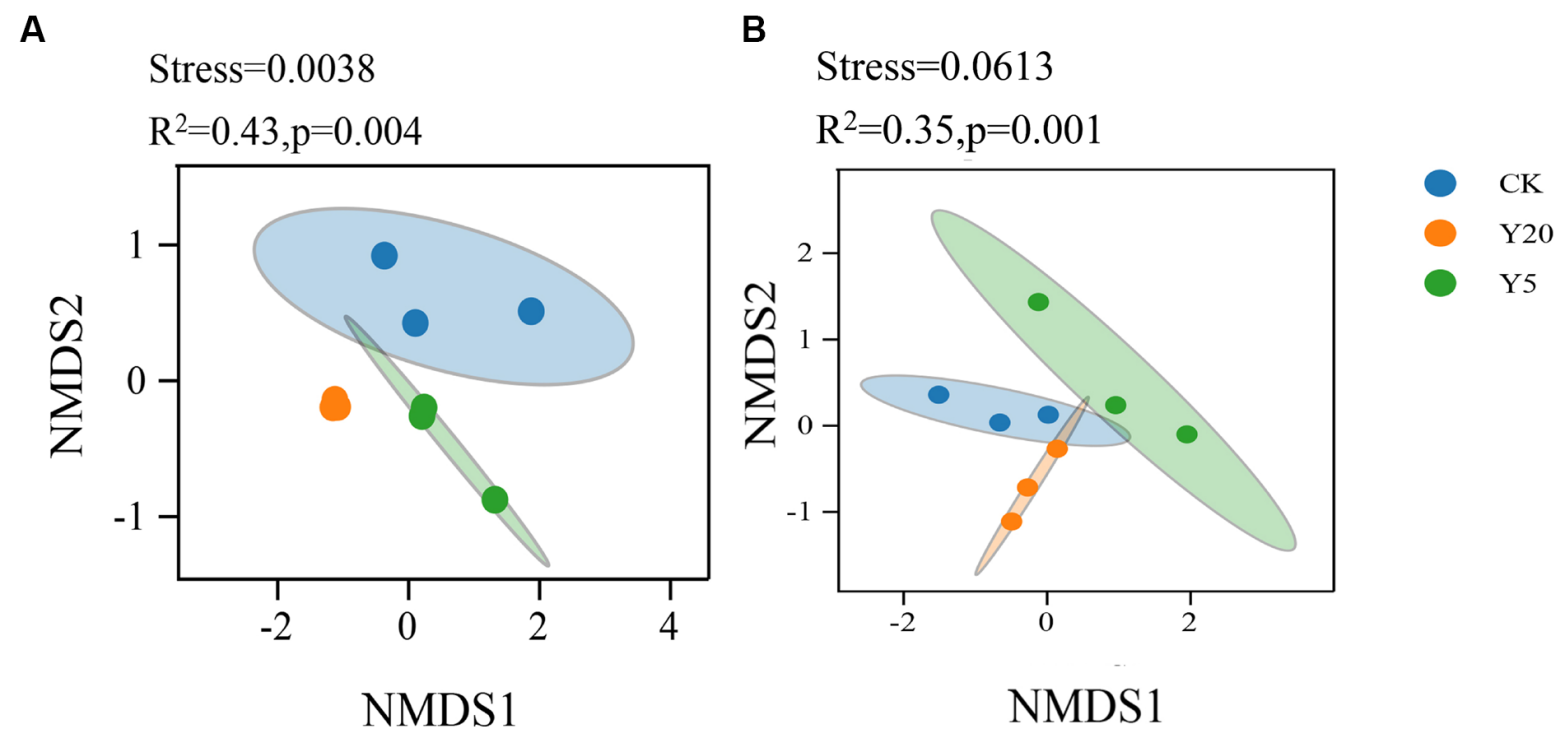
Non-metric multidimensional scaling (NMDS) analysis based on Weighted uniFrac distance for the bacterial **(A)** and fungal **(B)** communities.

Similarly, with the process of time, the community structure of bacteria and fungi changed significantly (*R*^2^ = 0.43, *p* < 0.01; *R*^2^ = 0.35, *p* < 0.01) (Figure 2). Weighted UniFrac nonmetric multidimensional scaling (NMDS) analysis (stress = 0.001) showed that the bacterial community structure was significantly different between three groups. The phylogenetic structures of soil fungal communities varied after different continuous cropping durations, where the three groups were significantly distinctive from other groups.

### Environmental factors correlated to microbial communities

**Figure 3:**
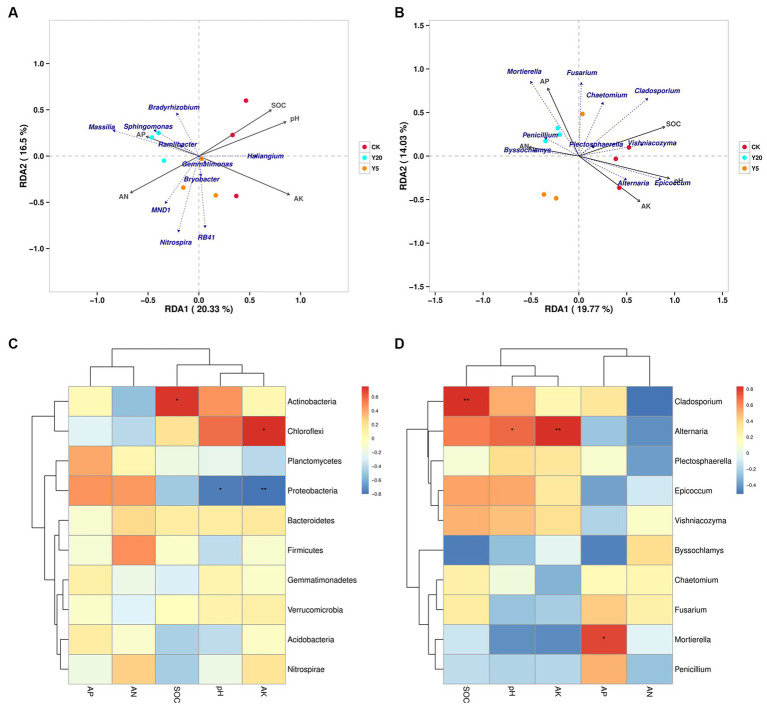
Redundancy analyses (RDA) based on bacterial **(A)** and fungal **(B)** OTU data with chemical parameters in soils with the lengthening of continuous cropping. Correlation heatmap of the top 10 bacterial **(C)** and fungal **(D)** genera with soil properties. *R* values are indicated on the right side of the legend with different colors. ^*^p < 0.05, ^**^p < 0.01, and ^***^p < 0.001.

Based on OTU reads and environmental factors, the representational difference analysis (RDA) was performed on the different continuous tobacco cropping. The relationships between bacterial communities and soil properties are shown in Figure 3A (axis 1 = 20.33%, axis 2 = 16.5%). The results indicated that the pH level (*R*^2^ = 0.8590, *p* = 0.008), SOC content (*R*^2^ = 0.7509, *p* = 0.013), and AK content (*R*^2^ = 0.9267, *p* = 0.003) were significantly correlated with bacterial communities. The correlation heatmap showed that at the phylum level, the abundance of Actinobacteria was significantly positively correlated with the SOC content (*p* < 0.05), and the abundance of Chloroflexi was significantly positively correlated with the AK content. In addition, the abundance of Proteobacteria was significantly negatively correlated with the pH level and the AK content. At the genus level, the abundance of *Micromonosporaceae* was positively correlated with the pH level and the AK content, and the abundance of *Acidobacteriales* was positively correlated with the AN content (*p* < 0.05) (Figure 3C). Furthermore, the abundance of *Massilia* was negatively correlated with the pH and AK content.

Figure 3B (axis 1 = 19.77%, axis 2 = 14.03%) showed the relationships between fungal communities and soil properties. The pH level (*R*^2^ = 0.957, *p* = 0.001), SOC content (*R*^2^ = 0.842, *p* = 0.004), AP content (*R*^2^ = 0.6582, *p* = 0.041), and AK content (*R*^2^ = 0.671, *p* = 0.037) were the main factors affecting the changes in fungal phylogenetic structure (Figure 3B). The abundance of Chytridiomycota was significantly positively correlated with the pH level and the AK content (*p* < 0.01), the abundance of Ascomycota was significantly positively correlated with the SOC content (*p* < 0.001), and the abundance of Mortierellmycota was significantly positively correlated with the AP content (*p* < 0.05). In addition, the abundance of Glomeromycota was significantly negatively correlated with the AP content, and the abundance of Olpidiomycota was significantly negatively correlated with the AN content. At the genus level, the abundance of Alternaria was positively correlated with the pH level and the AK content, and the abundance of Cladosporium was positively correlated with the SOC content (*p* < 0.01) (Figure 3D). Moreover, the abundance of Mortierella was positively correlated with the AP content (*p* < 0.05).

### Soil microbial function prediction

**Figure 4:**
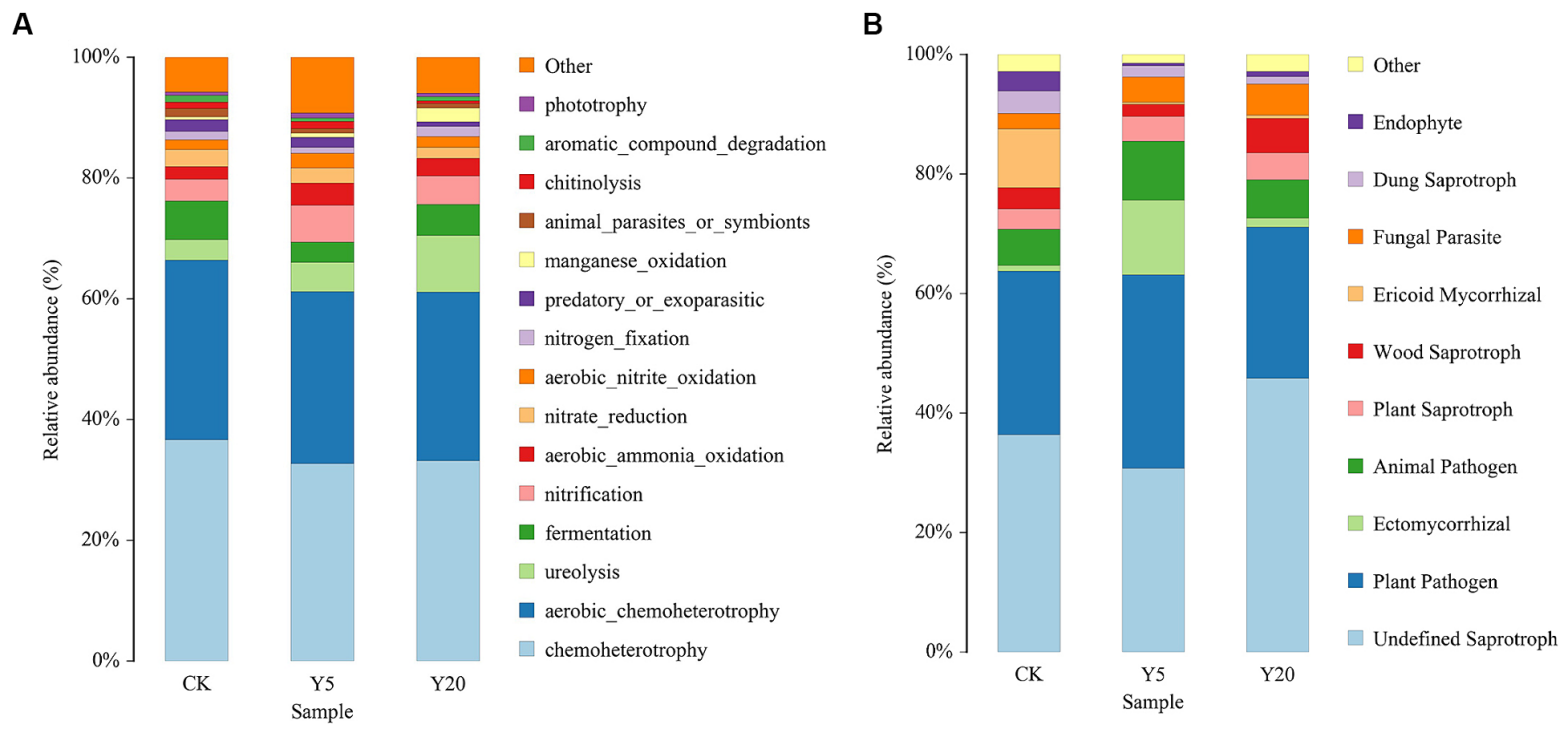
Changes in functional groups on bacterial **(A)** and fungi **(B)** OTU data in different continuous tobacco cropping.

Based on the FAPROTAX database, we predicted the bacterial functions and obtained the top 15 functional groups. The results showed significantly enhanced ureolysis and manganese oxidation, while nitrate reduction, predatory or ectoparasitic, and chitinolysis were significantly weakened (Figure 4A). Other functional groups showed no significant changes, such as chemoheterotrophy, aerobic chemoheterotrophy, aerobic ammonia oxidation, fermentation, nitrogen fixation, and nitrification.

To study the functional difference of fungal communities in samples, FUNGuild database was used to perform a functional prediction (Figure 4B). The fungal community trophic types included pathotrophs, saprotrophs, and symbiotrophs. Saprotrophs was dominated in CK accounting for 36% with plant pathogen accounting for 26% of the total community. Plant-pathogens were increased and then decreased with the duration of continuous cropping. Ectomycorrhizal were significantly increased in Y5 accounting for 13% compared to all the other treatment groups.

### Analysis of metabolites in soil

**Figure 5:**
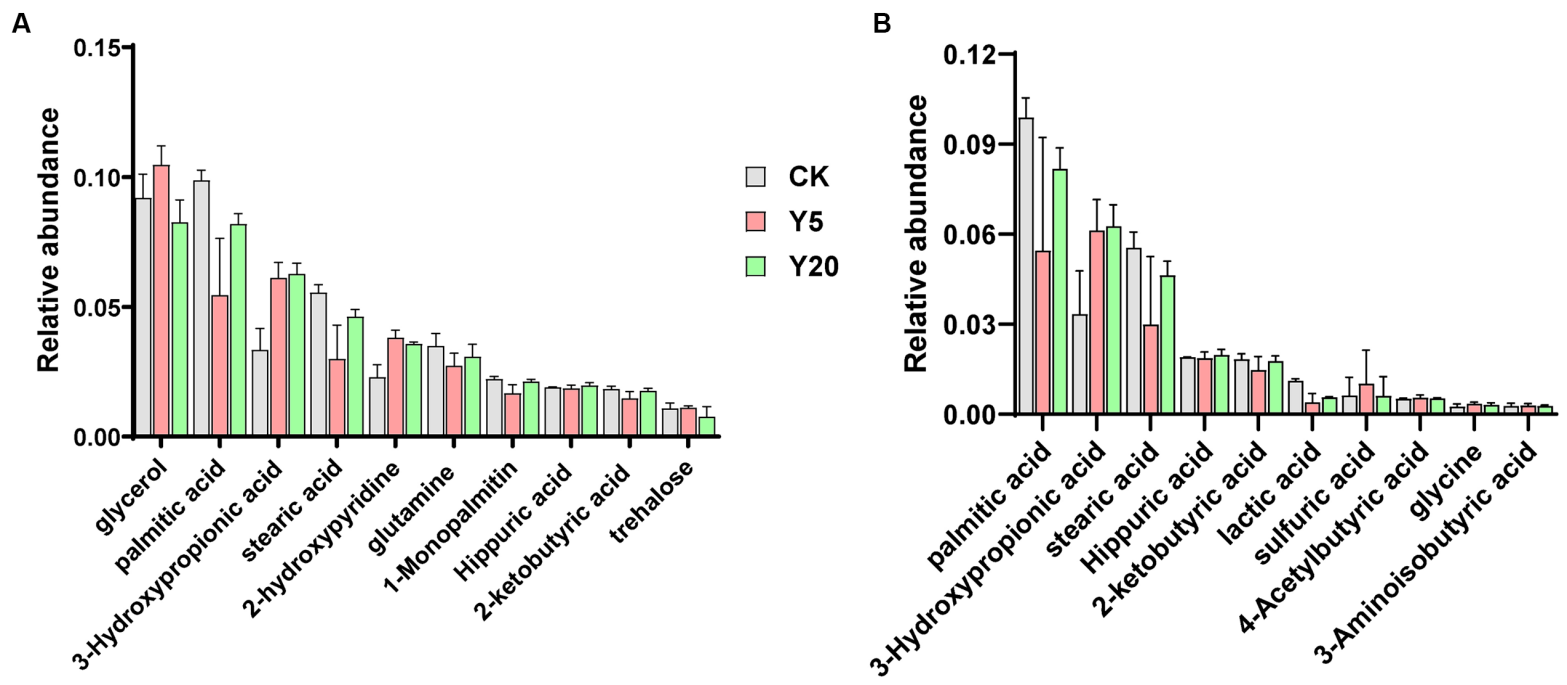
The relative abundances of metabolites **(A)** and acid compounds **(B)** in different soils with the lengthening of continuous cropping.

A total of 823 metabolites were detected through GC–MS, and 99 were identified. The total ion mass spectra of soil in CK, Y5, and Y20 were basically similar in profile and in peak time. The results indicated that the types of metabolites were similar in different continuous cropping duration. Among the top 30 compounds in relative abundance of metabolites, 14 acids, five alcohols, three sugars, two esters, two phenols, and six other complex compounds were detected (Figure 5A). As shown in Figure 5B, we observed that the samples contained acidic component as palmitic acid, 3-hydroxypropionic acid, stearic acid, hippuric acid, 2-ketobutyric acid, lactic acid, 4-acetylbutyric acid, glycine, 3-aminoisobutyric acid, citrulline, sulfuric acid, citraconic acid, valine, and cycloleucine. With the lengthening of continuous cropping, the content of 3-hydroxypropionic acid increased significantly, and that of palmitic acid and stearic acid decreased rapidly and then increased significantly. The other compounds showed no significant change in CK, Y5, and Y20.

**Figure 6:**
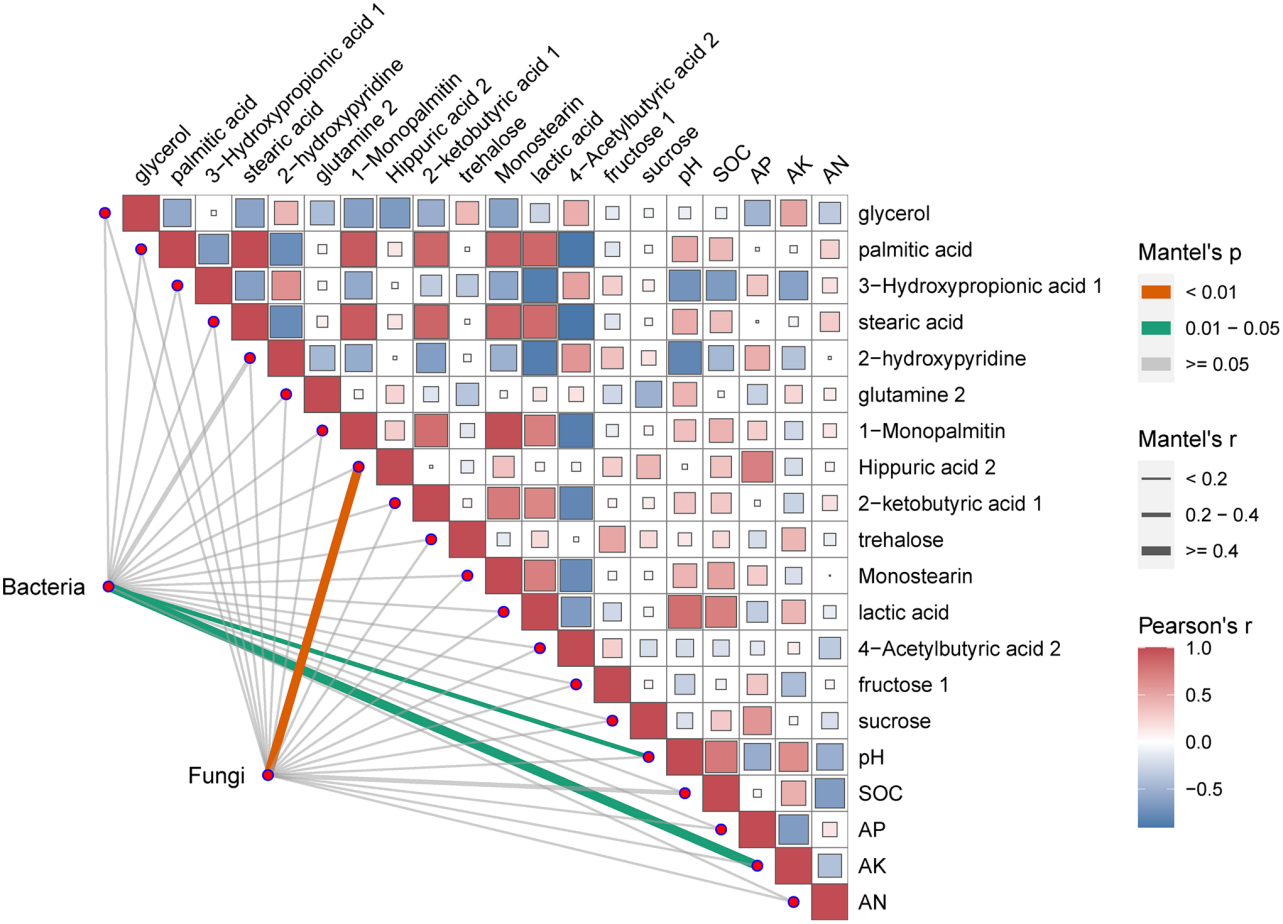
Effects of environmental variables on soil microbial diversity in different soils.

One trait influencing rhizosphere microbial changed could be soil micro-environment. To address this hypothesis, a correlation analysis between the rhizosphere microorganisms whose abundance markedly changed at the genus level and metabolites in soil was performed. The results of the correlation analysis were shown in Figure 6. The results revealed that pH and AN significantly correlated with bacteria. Meanwhile, 1-Monopalmitin significantly correlated with fungi.

## Discussion

Soil microbial communities are essential for plant nutrition and soil health (Fitzpatrick et al., 2018). However, long-term continuous cropping system breaks the balance of soil microbial community structure. Few scholars have evaluated the effects of long-term continuous cropping in karst agroecosystems of Guizhou province, China. Continuous cropping mode may decrease the nutrient ratio of soil and increase the incidence of pests, leading to poor crop growth and decreased yield and quality (An et al., 2022). The extensive use of pesticides brings safety issues and soil environmental pollution during agricultural production and seriously harms the health of soil ecosystems (Jiang et al., 2022). However, information on the ecological interactions between soil microbiomes and the establishment of disease-suppressive systems is still scarce. Therefore, clarifying the impact of long-term continuous cropping on karst agroecosystems may provide new avenues for managing microbiome-driven soil self-recovery.

### Soil microbial community changes

High-throughput sequencing revealed that bacterial community increased with the increase in continuous cultivation years, consistent with the findings of some studies on other crop species (Zhang et al., 2017; Fei et al., 2021; Jin et al., 2021; Yao et al., 2023). Specifically, the bacterial community composition was dominated by Proteobacteria, Acidobacteria, Actinobacteria, and Gemmatimonadetes at the bacterial phylum level. The abundance of Proteobacteria was increased, which may change the ecological function of soil. Proteobacteria are a broad phylum and can grow fast in soils with unstable substrate conditions (Zeng et al., 2017). The increase in easily decomposable nutrients can stimulate the increase in the abundance of Proteobacteria. Acidobacteria have a wide range of metabolic and genetic functions and exhibit a robust inverse response to soil pH (Wang et al., 2016). Interestingly, the abundance of Acidobacteria increased and then decreased as soil pH decreased in our study. Copiotrophic microbes are regarded to be related to R-strategy (Fierer et al., 2007). The theory of microbial nutrition strategies assists in comprehending our results. In our study, the relative abundances of *Sphingomonas* and *Massilia* were significantly higher in Y20 than in other groups, whereas those of *Gemmatimonas*, *Micrococcaceae*, and *Micromonosporaceae* showed the opposite trends. *Sphingomonas* can survive under nutrient conditions and is essential in decomposing recalcitrant organic matters. It has exploitable potential in ecological environment restoration and will be a hot spot in the microbial restoration of the environment in the future. *Massilia* presents in rhizosphere soil as symbiotic bacterium (Cardinale et al., 2019). In this study, the RDA results indicated that the AP content was positively correlated with the relative abundances of *Sphingonas* and *Massilia* (Figure 3), and the changes in AN content could indirectly affect their relative abundances. Previous reports have shown that *Sphingomonas* and *Massilia* can dissolve phosphorus, and their relative abundances are positively correlated with the activity of phosphatase (Cardinale et al., 2019; Son et al., 2021). We speculate that the increases in the relative abundances of *Sphingomonas* and *Massilia* provide more AP for plants and fungi in continuous cropping soil. *Gemmatimonas*, *Micrococcaceae*, and *Micromonosporaceae* are beneficial bacteria that widely exist in soil and play a very important role in the stability of soil ecosystems. In this study, the abundances of *Gemmatimonas*, *Micrococcaceae*, and *Micromonosporaceae* decreased significantly with continuous cropping. We speculated that tobacco roots continuously secreted secondary metabolites, contributing to the colonization of certain microbial species whereas inhibiting that of certain beneficial microbial species in continuous cropping systems (You et al., 2015; Zhang et al., 2020).

We observed that different shifts in soil fungal compositions across different durations of continuous cropping. The abundance and diversity of fungal communities increased with the lengthening of continuous cropping in alpha diversity. The dominant genera in all groups were *Mortierella*, *Fusarium*, *Byssochlamys*, *Chaetomium*, *Penicillium*, and *Cladosporium*. Among them, *Mortierella* is a beneficial fungus with contributions to soil nutrient transformation and availability (Ning et al., 2020). Adding *Mortierella* to soil can significantly increase the contents of AP, potassium, AK, magnesium, and boron (Francioli et al., 2016). *Chaetomium* is an important fungus with a biocontrol effect, contributing to the decomposition of cellulose and residing within various plant residues and soils (Hubbard et al., 2011). *Byssochlamys*, *Fusarium*, and *Cladosporium* are plant pathogens. *Fusarium* is a widely reported pathogenic fungus; plants infected with it can wilt, which will be exacerbated by continuous cropping (Sant'Ana et al., 2009; Wang et al., 2015). Mildly acidic conditions are suitable for the growth of *Byssochlamys*, which has a broad host spectrum and is pathogenic to many plants (Sant'Ana et al., 2009). The relative abundance of *Fusarium* tends to decrease and increase subsequently with the lengthening of continuous strawberry cropping (Huang et al., 2018). In this study, the relative abundances of potential phytopathogens (*Byssochlamys* and *Fusarium*) decreased and then increased with the duration of continuous cropping. Continuous soybean cropping increases the relative abundance of beneficial fungi, such as *Mortierella* sp., to inhibit plant pathogens (Liu et al., 2020). In this study, the relative abundance of the pathogenic fungus *Mortierella* was increased with the duration of continuous cropping. We speculated that tobacco root exudates recruited beneficial fungi to compete with pathogenic microbiomes under long-term continuous cropping. Overall, potential plant pathogenic fungi and beneficial microorganisms showed synergistic increases under long-term continuous cropping.

### Soil microbial community response to environmental drivers

Previous studies have shown that long-term continuous cropping leads to the deterioration of soil chemical properties and decreased plant yields (Liu Q. et al., 2021). Continuous cropping obstacles may be associated with changes in soil pH and nitrogen content. The decreased pH level reduces the availability of soil nutrients and interferes with nutrient absorption, impeding normal crop development (Huang et al., 2018). Continuous cropping obstacles may be attributable to the decreased soil pH (Zhang et al., 2015). This study demonstrated that the soil pH level was negatively associated with the AN content, which agrees well with a previous study (Zhang and Lin, 2009). The contents of AP and AN after continuous soybean cropping for 13 years were significantly higher than those after 3 or 5 years. Soil nutrients exhibited no decrease after long-term continuous cropping, indicating that the lack of plant nutrients may not directly cause plant diseases (Mbanyele et al., 2022).

Furthermore, soil microbial communities were affected by environmental factors, including soil pH levels and the contents of AN and AP. The RDA and correlation heatmap were used to analyze the relationships between soil microbial composition and environmental factors (Figure 3). Previous studies have demonstrated that environmental factors have different effects on bacterial and fungal communities (Dong et al., 2017). This study found that the effects of environmental factors on bacterial diversity were more dramatic than fungal diversity. Among the bacteria, the abundance of *Micromonosporaceae* was positively correlated with the pH level and AK content, that of *Acidobacteriales* was positively correlated with the AN content, and that of *Massilia* was negatively correlated with the pH and AK content. Among the fungi, the abundance of *Alternaria* was positively correlated with the pH and AK content, that of *Cladosporium* was positively correlated with the SOC content, and that of *Mortierella* was positively correlated with the AP content. In summary, the pH level and AK content had the greatest influence on the soil microbial community structure, while the AP content significantly influenced the formation of the fungal community structure.

### Soil microbial function predictions

Different microbial functional communities were formed at different successional stages (Liu S. et al., 2021). The abundances of different bacterial metabolic pathways were predicated using the FAPROTAX database. We observed significant increases in ureolysis and manganese oxidation and significant decreases in nitrate reduction, predatory or ectoparasitic, and chitinolysis with the duration of continuous cropping. According to the nutrient competition hypothesis, the growth and reproduction of mycorrhizal fungi and free microorganisms need nutrients (Orwin et al., 2011). Previous studies have reported that ectomycorrhizal and plant pathogens can secrete enzymes to degrade organic matter, which inhibits the nitrogen restriction of free microorganisms, thus inhibiting organic matter decomposition (Cederlund et al., 2014; Averill, 2016). We found that the abundances of plant-pathogens and ectomycorrhiza increased and then decreased, which may be caused by the interactions of ectomycorrhizal and plant pathogens with free microorganisms. Finally, we concluded that fungal changes might alter the soil nutrient cycles in karst ecosystems under long-term continuous cropping.

### Analysis of metabolites in soil

The deterioration of soil chemical properties was considered to be associated with long-term continuous cropping crops (Xie et al., 2017). It was reported that crop released a great variety of secondary metabolites into the soil environment through root exudation or residue decomposition (Liu et al., 2018). The allelochemicals have been proposed that can improve the activities of soil-borne pathogens, which are released by the crops (Bonanomi et al., 2016). In our finding, the soil pH decreased significantly with the duration of continuous cropping increased, which is consistent with previous studies (Chen et al., 2022). The accumulation of acids might cause soil acidification (Agegnehu et al., 2016). There had differences in metabolites in soil among different periods of continuous cropping in this finding. The content of palmitic acid and stearic acid increased initially and then decreased as the cropping continued. The palmitic acid influenced plant growth and metabolism, which have found in high levels in many plant root exudates and residues (Ma et al., 2021; Xin et al., 2022). Previous studies have proved that palmitic acid and stearic acid can significantly inhibit the germination of seeds and the growth of seedlings (Xiang et al., 2022). In the process of penetration and colonization, fungi can secret an array of extracellular hydrolytic enzymes, which can degrade cell walls of roots, secreting substantial quantities of organic acids (Gu et al., 2022). In this study, we used GC-TOF-MS to analyze secondary metabolites in soil and found many organic acids and their derivatives. Whether those metabolites are root exudates or secreted by microorganisms remains to be further verified. In addition, our work has some limitations, and additional studies are needed to investigate the dynamic changes in soil metabolites and their relations with soil microorganisms. These finding could be used to reveal the mechanism of soil sickness associated with long-term continuous cropping crops.

## Conclusion

In this study, we demonstrated that continuous cropping system exerted a significant impact on the microbial community structure, exhibiting discernible disparities between long-term and short-term continuous cropping farmland. Furthermore, the soil pH level as well as AN content and SOC content were key environmental factors contributing to microbial community structural and functional changes. Long-term continuous cropping fostered synergistic increments in potential plant pathogenic fungi and beneficial microorganisms. However, further research is warranted to investigate whether and how pathogenic and beneficial microorganisms interact with plant secondary metabolites requires.

## Data availability statement

The datasets presented in this study can be found in online repositories. The names of the repository/repositories and accession number(s) can be found in the article/Supplementary material.

## Author contributions

MD: Conceptualization, Data curation, Writing – original draft. HD: Writing – review & editing, Conceptualization, Validation. YH: Funding acquisition, Methodology, Validation, Writing – review & editing. TL: Formal analysis, Validation, Writing – review & editing. ZZ: Formal analysis, Supervision, Writing – review & editing. SZ: Project administration, Supervision, Writing – review & editing. BH: Writing – review & editing, Data curation, Formal analysis. HC: Writing – review & editing, Data curation, Methodology. BD: Methodology, Validation, Writing – review & editing. YX: Writing – review & editing, Formal analysis, Investigation. YZ: Funding acquisition, Supervision, Writing – review & editing.
