# Supplementary material for: Continuous cropping system altered soil microbial communities and nutrient cycles
Source: Front Microbiol. 2024 Apr 12;15:1374550. doi: 10.3389/fmicb.2024.1374550 (PMC11045989; doi:10.3389/fmicb.2024.1374550)
Supplement: Supplementary file 1 [file Data_Sheet_1.pdf]

Environmental factor	Bacterial community				Fungal community			
	R^2	*P*-value	Unique	I.perc(%)	R^2	*P*-value	Unique	I.perc(%)
pH	0.859	0.008	0.061	31.90	0.957	0.001	0.064	65.11
SOC	0.751	0.013	0.123	10.69	0.842	0.004	0.206	83.02
AP	0.300	0.327	0.06	-6.17	0.658	0.041	0.065	16.04
AK	0.927	0.003	0.287	27.27	0.671	0.037	0.031	0.50
AN	0.604	0.069	0.311	36.31	0.278	0.405	-0.042	-32.88

Supplemental Table S1. Redundancy analysis (RDA) of effects of different environmental factors on soil microbial community. Environmental factors including pH, soil organic carbon (SOC), available phosphorus (AP), available potassium (AK), and alkeline nitrogen (AN). The relative importance of each explanatory variable independently accounting for total variations was quantified for the total variations was qualified by applying the hierarchy algorithm. Unique: each explanatory variable independently accounting for total variations. I.perc(%): individual effect divided by total adjusted R^2 found in column Individual importance.

Supplemental Table S2. Alpha diversity indexes of bacterial and fungal communities under long-term continuous cropping.

	Bacteria				Fungi			
Treatment	Shannon	Simpson	Ace	Chao1	Shannon	Simpson	Ace	Chao1
CK	8.798a	0.990a	1774.719a	1805.053a	5.398a	0.931a	466.179a	485.739a
Y5	8.961a	0.992a	1842.663a	1879.089a	5.151a	0.924a	513.141a	531.254a
Y20	8.744a	0.989a	1869.586a	1902.928a	5.496a	0.951a	463.895a	482.369a

CK: tobacco cropping for 0 years. Y5: continuous tobacco cropping for 5 years. Y20: continuous tobacco cropping for 20 years. Different letters indicate significant differences among the three treatments tested by one-way ANOVA ($P < 0.05$).

Supplemental Figure S1. Major variations in the bacterial (A) and fungal (B) communities in different continuous tobacco cropping soil detected by the PCA of the OTUs.
