# Supplementary figures and images for: Continuous cropping system altered soil microbial communities and nutrient cycles
Source: Front Microbiol. 2024 Apr 12;15:1374550. doi: 10.3389/fmicb.2024.1374550 (PMC11045989; doi:10.3389/fmicb.2024.1374550)

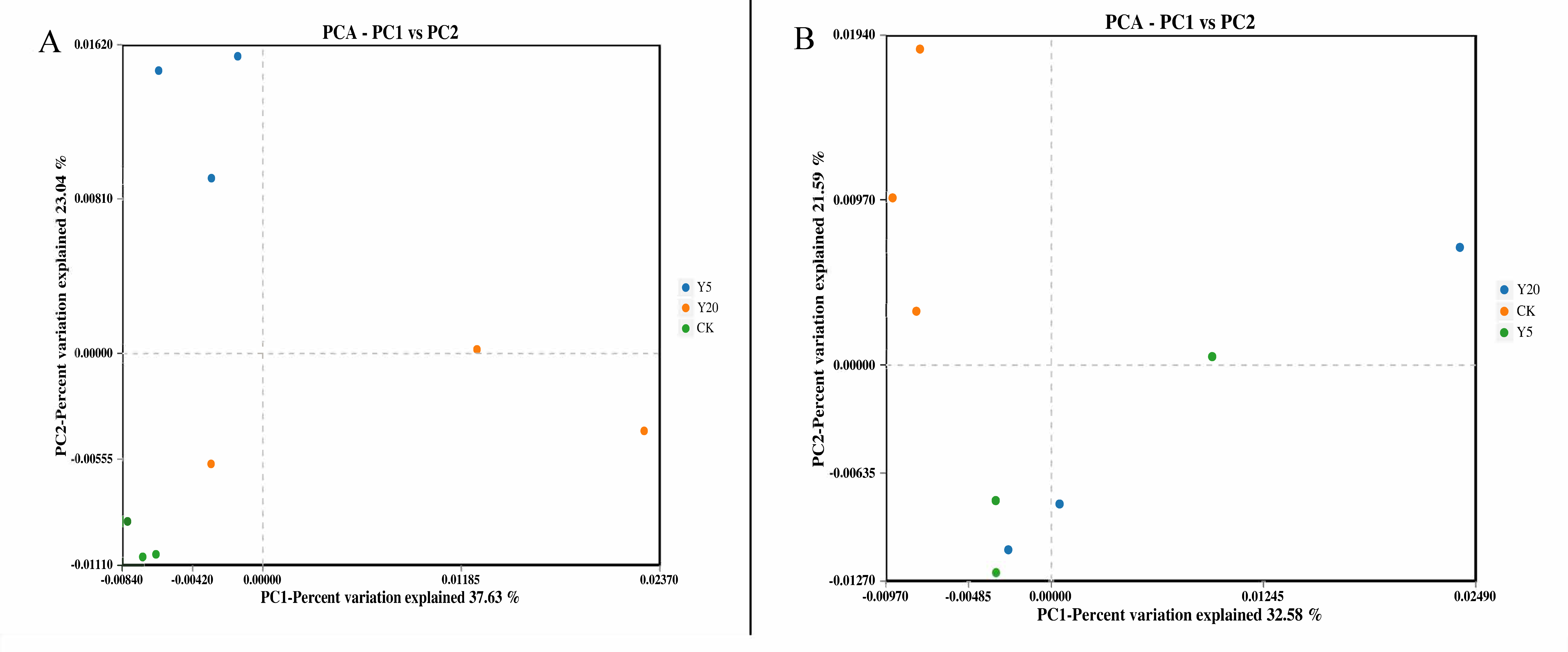

Supplement: Supplementary file 2 [file Image_1.JPEG]
